# Morphometric analysis of odontoid process among Arab population: a retrospective cone beam CT study

**DOI:** 10.7717/peerj.15411

**Published:** 2023-05-23

**Authors:** Asmaa Uthman, Basheer Salman, Hawraa Shams Aldeen, Hesham Marei, Sura F. Al-Bayati, Natheer H. Al-Rawi

**Affiliations:** 1 Department of Diagnostic and Surgical Dental Sciences, College of Dental Medicine, Gulf Medical University, Ajman-Al-Jurf, Ajman, United Arab Emirates; 2 Department of Oral and Craniofacial Health Sciences, College of Dental Medicine, Sharjah, United Arab Emirates

**Keywords:** Odontoid process, CBCT, Morphometry

## Abstract

**Objective:**

This study aims to evaluate the feasibility of using cone beam computed tomography (CBCT) scans to assess the odontoid process diameter in the Arab population and to determine whether one or two cortical screws can be used for treating odontoid fractures.

**Methods:**

The odontoid process of 142 individuals aged 12–75 years, including 72 males (mean age: 35.5) and 70 females (mean age: 36.2), were analyzed using CBCT scans. The sagittal and coronal CBCT views were used to evaluate the antero-posterior (AP) and transverse diameters of the odontoid process.

**Results:**

Males had substantially bigger transverse and AP diameters of the odontoid process than females (*p* < 0.05 & *P* < 0.01 respectively). Among the sample, 97 individuals (67.4%) had external transverse diameter (METD) of less than 9 mm which is slightly bigger than that of Indians and 48 individuals (31.83%) had enough room for two 3.5 mm or two 2.7 mm screws as their METD was more than 9 mm like that of Greek and Turkish. Age had no significant impact on the morphometric measurements of the odontoid process.

**Conclusion:**

More than sixty percent of the sample had METDs of less than 9 millimeters, indicating that a single 4.5-mm Herbert screw may be suggested for fixing fractured odontoid processes in the Arab population.

## Introduction

The human neck is supported by seven cervical vertebrae, which comprise the most superior segment of the spinal column (Hiatt, 2010). These vertebrae are located between the rib cage and the skull (Moore & Dalley, 2022). The atlas and axis, the top two cervical vertebrae, have undergone significant changes to enable the head to rest on them and rotate on the spinal column at the atlanto-axial joint (Madawi et al., 1997). The odontoid process, or dens, is a conical structure that protrudes about 1.5 cm cranially from the body of the axis, and it is distinctive from the rest of the spinal column due to its anterior location to the spinal cord and its role in anchoring the cranio-vertebral junction (CVJ) (Moore & Dalley, 2022; Lalit et al., 2019; Susan, 2020; White, Black & Folkens, 2011; Bambakidis & Dickman, 2012). The dens can be off-center laterally up to 10 degrees or posteriorly up to 14 degrees from the axis’s body (Susan, 2020). Congenital or acquired conditions can cause instability of the atlantoaxial joint, resulting in neurological symptoms, and differences in odontoid process architecture (Akobo et al., 2015). Treatment options for odontoid fractures range from restorative to surgical, with surgical options including posterior C1-C2 fusion, anterior osteosynthesis with a plate and screws, or repair of the odontoid fracture with one or two screws (Korres, 2013). Accurate radiographic examination and assessment of odontoid morphometric parameters are crucial for stable fixation and fusion of odontoid fractures (Korres et al., 2016). Odontoid fractures represent 50–60% of C2 fractures, 7–27% of cervical vertebral column fractures, and 1–2% of total vertebral column fractures (Naderi et al., 2006; Montemurro et al., 2022). Screw placement indications differ from fracture to fracture and odontoid process to odontoid process, and the insertion space limits may make it difficult to use two 3.5 mm screws  (Bohler, 1982). The precise diameter of the odontoid process is critical as it varies from person to person (Daher et al., 2011; Nucci et al., 1995; Yusof et al., 2007), and cervical spinal fractures are more common in individuals who have been in car accidents in the Arab population (Grivna, Eid & Abu-Zidan, 2015). Several imaging modalities that can be used to visualize the odontoid process, like X-ray which shows the odontoid process and surrounding bones in two dimensions. Multiple detector CT (MDCT) & CBCT scans also shows the odontoid process and adjacent bones in 3D. This imaging method can detect bone fractures (Keller et al., 2015; Shin et al., 2022). Magnetic resonance imaging (MRI): This imaging technique detects ligament and soft tissue injury. In this study, we aim to use cone beam computed tomography (CBCT) to assess the odontoid process diameter in the Arab population and evaluate the feasibility of treating odontoid fractures with one or two cortical screws.

## Patients and Methods

### Study design and study population

Between January 2012 and December 2016, a retrospective analysis was conducted on 142 cone beam computed tomography (CBCT) scans of patients who had visited the University Dental Hospital, Sharjah (UDHS) for various dental procedures. The study was approved by the human subject’s ethics board at the University of Sharjah (REC-19-01-24-01), and it was conducted in compliance with the Helsinki Declaration of 2014 (World Medical Association, 2013).

Using G power analysis software with the effect size set to 0.5, the error probability set to 0.05, and the power of the study set to 0.9, a priori sample size of 140 was established. The scans were obtained using a Galileo’s Comfort CBCT machine (Sirona Dental Systems, Bensheim, Germany), with imaging parameters of a 17 × 13 cm field of view, 85 kVp, 7 mA, 14 s exposure, and 0.25 mm voxel size. The CBCT images were displayed on a 23-inch, 1920 × 1080-pixel DELL monitor using SIDEXIS software. Two experienced dental radiologists (AU and HS) analyzed the CBCT images, and in the event of any discrepancies between the two primary examiners, a third examiner (NA) with similar experience was consulted for review. Intra-observer reliability was assessed by having the same radiologists reexamine the scans after 15 days.

### Inclusion criteria

Including only high-quality images into the research is crucial for obtaining accurate measurements of the odontoid process. Age-appropriate gender balance is also important; thus the sample should include both men and women of similar ages.

### Exclusion criteria

Fractures, tumors, or infections of the dens, as well as those whose radiological scans failed to meet the predetermined measurement standards or whose dynamic CBCT scans produced subpar images, were all reasons to exclude participants from the research. The study comprised 72 male and 70 female participants, with a mean age of 39 and 36 years, respectively, and an age range of 12–75 years.

### Definition of anatomical landmarks used

All measurements were obtained using the CBCT scan software, with manual measurements being taken in the sagittal and coronal views perpendicular to the long axis of the odontoid process at 0.8 mm intervals. The base of the odontoid process was identified as the lowest level where it was most clearly delineated as described by Korres et al. (2016).

**Figure 1:**
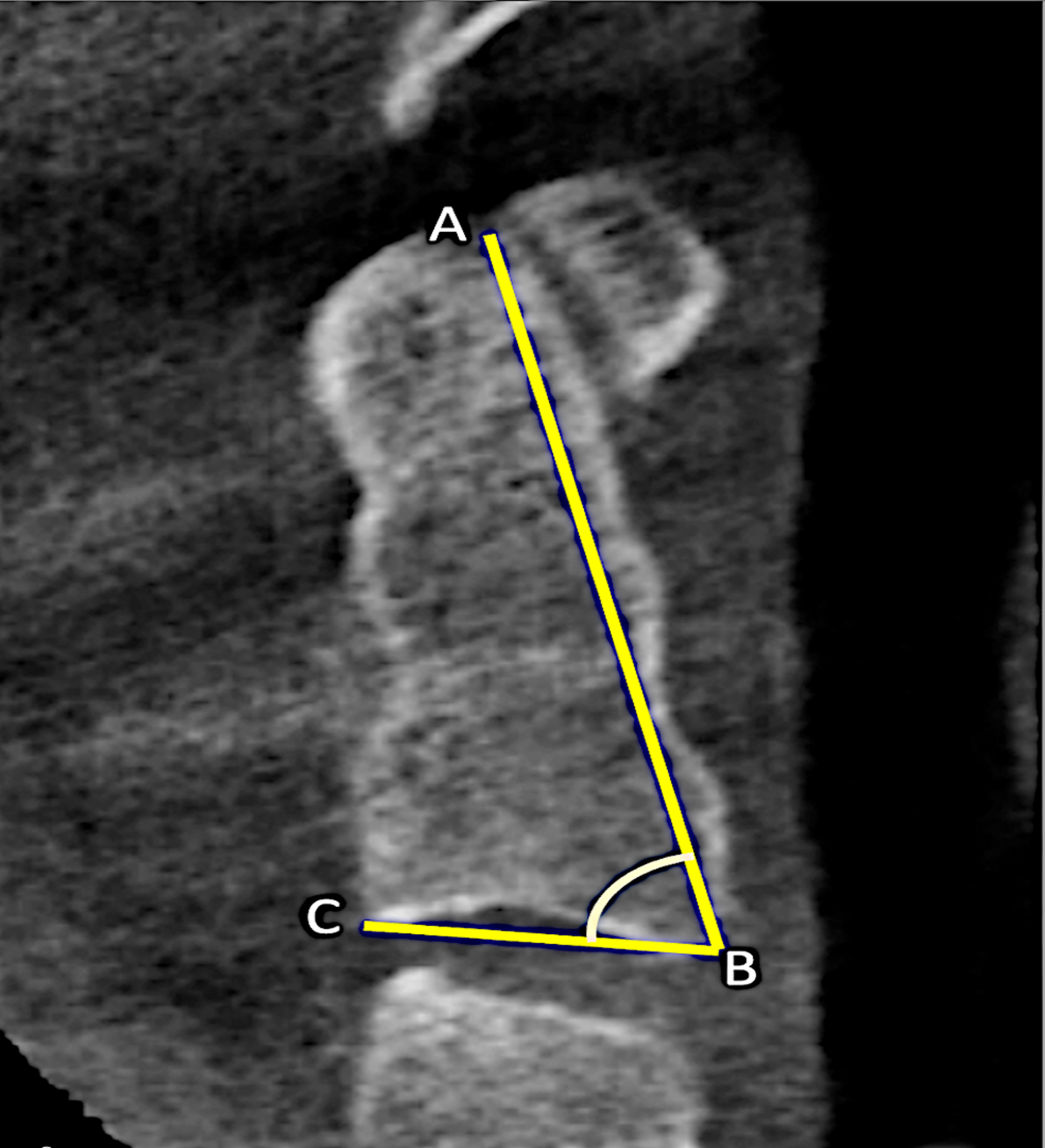
Morphometric measurements of of odontoid process length (AB) and angle (ABC).

**Figure 2:**
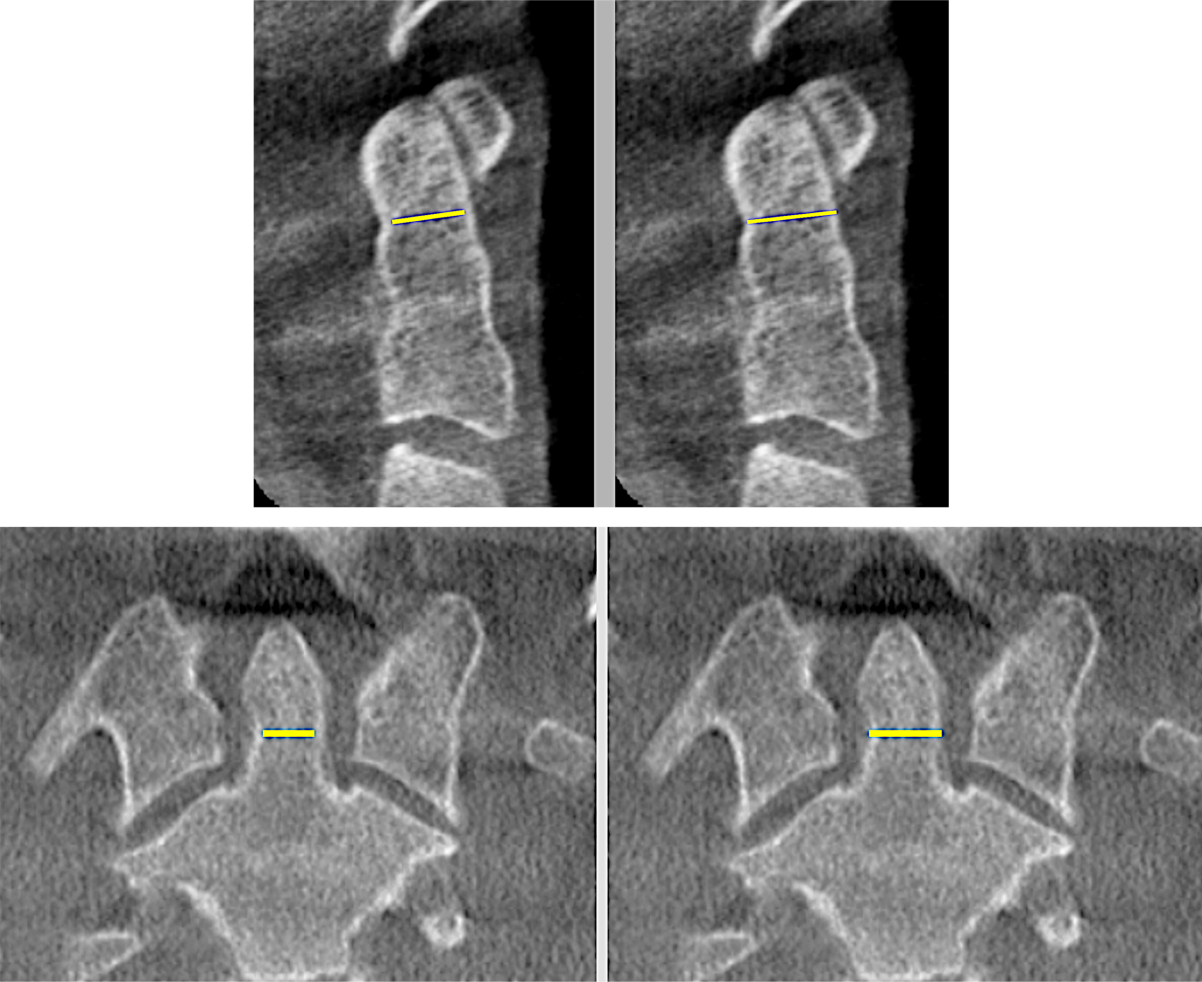
Measurement of the internal diameter of odontoid process from interior cortex outline at the neck of odontoid in the sagittal view. (MIAP) (top left), Measurement of the external diameter of odontoid process from exterior cortex outline at the neck of odontoid in the sagittal view (MEAP) (top right). Measurement of the internal transverse diameter of odontoid process from interior cortex outline at the neck of odontoid process in the coronal view (MITD) (bottom left). Measurement of the external transverse diameter of odontoid process from exterior cortex outline at the neck of odontoid in the coronal view (METD) (bottom right).

From sagittal view, the height and angle of the odontoid process were measured (Fig. 1). The neck of the odontoid process was determined to be the dens’ lowest (well-looking) level. The slices were obtained at 0.8 mm intervals from the base of the odontoid process. The neck of the odontoid process is the lowest level with the most distinct appearance of dens. Measurements were taken of the anterior–posterior external diameter (MEAP), anterior–posterior internal diameter (MIAP), transverse external diameter (METD), and transverse internal diameter (MITD) (Figs. 2A–2D).

After identification the slice of interest, we used the magnification tool in the imaging software to better visualize the bone cortex outline. The measurements were based on the visualized cortical bone outline interiorly and exteriorly.

Table 1 lists the CBCT variables measured and evaluated in this investigation.

### Statistical analysis

Once the normal distribution of the variables was confirmed, the independent sample Student’s *t*-test was employed to compare the variables between genders, while the ANOVA test was utilized to examine the differences among different age groups. The Pearson correlation coefficient was employed to assess the relationships between the parameters under investigation. A *P* value less than 0.05 was considered significant.

## Results

### Inter and Intraclass (ICC) correlation

The inter-rater reliability of the linear measurements conducted by the two radiologists (AU and HS) yielded an ICC of 0.90, which indicates a high level of consistency. To assess intra-rater reliability, each examiner reevaluated 5% of the total sample 15 days after the initial evaluation, resulting in an ICC of 0.95. These findings suggest that the measurement method utilized in this study is highly reliable.

### Mean age differences between genders

There was no statistically significant difference observed in age between females and males, indicating that both genders were comparable in terms of baseline characteristics, as illustrated in Table 2.

**Table 1:** Morphometric variables of the odontoid process of C2 cervical vertebra from the sagittal and coronal CBCT views.

AB	The distance between the apex of the odontoid process and anterior border of the axis base (Fig. 1).
ABC	Angle between the line of the apex of the odontoid process to the anterior edge of the axis and tangent to the plateau below the axis (Fig. 1).
MIAP	Smallest measurement of internal diameter of odontoid process from interior cortex outline at the neck of odontoid in the sagittal view (Fig. 2 top left).
MEAP	Smallest measurement of external diameter of odontoid process from exterior cortex outline at the neck of odontoid in the sagittal view (Fig. 2 top right).
MITD	Smallest measurement of internal transverse diameter of odontoid process from interior cortex outline at the neck of odontoid process in the coronal view (Fig. 2 bottom left).
METD	Smallest measurement of external transverse diameter of odontoid process from exterior cortex outline at the neck of odontoid in the coronal view (Fig. 2 bottom right).

**Table 2:** Age and gender differences among odontoid variables.

**Variable**	**Males (*n* = 72)**		**Females (*n* = 70)**		***t* value**	***P* value**
	**Mean ± SD (mm)**	**Range**	**Mean ± SD (mm)**	**Range**		
Age	38.51 ± 17.34	11–75	36.20 ± 16.75	11–75	0.808	0.420
AB length	38.50 ± 3.34	31–46	35.59 ± 2.27	31–40	6.009	<0.001^**^
ABC angle	63.28 ± 3.94	53.50–75.10	62.48 ± 3.46	54.20–69.70	1.268	0.207
MIAP	8.43 ± 0.83	7–11	7.71 ± 0.80	6–10	5.249	<0.001^**^
MEAP	11.05 ± 0.77	9–13	10.13 ± 0.81	8–12	6.878	<0.001^**^
MITD	6.18 ± 0.78	5–8	5.87 ± 0.73	4–8	2.437	0.016^*^
METD	8.33 ± 0.75	6–10	7.97 ± 0.78	6–11	2.788	0.006^*^

**Notes.**

* Signifcant at *p* < 0.05.

** Significnat at *p* < 0.01.

### Gender differences among odontoid process variables

Table 2 shows that males had significantly greater values for MITD (6.18 ± 0.78 mm), METD (8.33 ± 0.75 mm), MIAP (8.43 ± 0.83 mm), MEAP (11.05 ± 0.77 mm), and AB (38.50 ± 3.34 mm) compared to females (5.87 ± 0.73 mm, 7.97 ± 0.78 mm, 7.71 ± 0.80 mm, 10.13 ± 0.81 mm, and 35.59 ± 2.27 mm, respectively). However, there was no significant difference in ABC angle between males and females.

### Age differences among odontoid process variables

To investigate how the morphometry of the odontoid process changes with time. The sample population was split into six groups, each representing a different age range by a ten-year threshold. According to Table 3, there were no appreciable variations in any of the odontoid process parameters between the young and old.

**Table 3:** Age differences among odontoid process variables.

**Variable**		**Age groups**						***F* value**	***P* value**
		**Gp 1 (<20 years) (*n* = 24)**	**GP 2 (20–29 years) (*n* = 30)**	**GP 3 (30–39 years) (*n* = 32)**	**GP 4 (40–49 years) (*n* = 16)**	**GP 5 (50–59 years) (*n* = 22)**	**GP 6 (>60 years) (*n* = 18)**		
**MITD**	**Mean ± SD**	5.77 ± 0.57	5.91 ± 0.79	6.04 ± 0.85	6.04 ± 0.77	6.34 ± 0.78	6.12 ± 0.742	1.453	0.210
	**Range**	5–7	4–8	4–8	5–8	5–8	5–8		
**METD**	**Mean ± SD**	7.94 ± 0.78	8.01 ± 0.74	8.18 ± 0.80	8.08 ± 0.71	8.38 ± 0.99	8.40 ± 0.53	1.322	0.259
	**Range**	6–10	6–9	6–11	6–9	6–10	7–10		
**MIAP**	**Mean ± SD**	7.81 ± 0.53	7.93 ± 0.78	8.18 ± 0.72	8.53 ± 0.95	8.07 ± 1.02	8.10 ± 1.33	1.533	0.184
	**Range**	7–9	6–9	6–11	6–9	6–10	7–10		
**MEAP**	**Mean ± SD**	10.14 ± 0.72	10.48 ± 0.85	10.70 ± 0.77	10.95 ± 0.75	10.66 ± 1.09	10.81 ± 1.19	2.141	0.064
	**Range**	9–11	9–12	9–12	10–12	9–12	8–13		
**ABC**	**Mean ± SD**	63.68 ± 4.13	63.18 ± 2.98	62.45 ± 3.95	64.68 ± 2.84	61.18 ± 4.15	62.61 ± 3.44	2.094	0.070
	**Range**	55.90–75.10	57.10–68.80	54.20–69.70	59.10–69.90	53.50–68.60	57.00–67.60		
**AB**	**Mean ± SD**	35.76 ± 3.14	36.79 ± 2.63	37.27 ± 2.41	38.01 ± 3.52	37.66 ± 4.15	37.40 ± 3.69	1.332	0.255
	**Range**	31–41	32–43	33–42	31–44	31–46	31–44		

### Correlation between different studied odontoid process variables in male and female groups

The results of the correlation analysis for males and females are presented in Table 4. Among males, there was a significant positive correlation between all odontoid process parameters, except for ABC with AB, which showed a significant negative correlation. The correlation coefficients (*r* values) and *p*-values for each correlation are presented in Table 4. The strongest correlation was observed between MITD and METD (*r* = 0.750, *P* < 0.01), followed by MIAP and MEAP (*r* = 0.704, *P* < 0.01). ABC showed no significant correlation with other odontoid parameters, except with AB. Among females, there was also a significant positive correlation between all odontoid process parameters, except for ABC with AB, which showed a significant negative correlation. The strongest correlation was observed between MIAP and MEAP (*r* = 0.795, *P* < 0.01), followed by MITD and METD (*r* = 0.763, *P* < 0.01). AI showed no significant correlation with other odontoid parameters, except with AB. Notably, the correlation between the studied variables was stronger in females than in males.

**Table 4:** Correlation between different studied odontoid variables in male and female group.

**Males**	**MITD**	**METD**	**MIAP**	**MEAP**	**ABC**	**AB**
MITD	1					
METD	0.750^**^	1				
MIAP	0.471^**^	0.306^**^	1			
MEAP	0.379^**^	0.384^**^	0.704^**^	1		
ABC	−0.062	−0.100	−0.001	−0.065	1	
AB	0.265^*^	0.271^*^	0.280^*^	0.458^**^	−0.337^**^	1
** Females**						
MITD	1					
METD	0.763^**^	1				
MIAP	0.468^**^	0.417^**^	1			
MEAP	0.420^**^	0.487^**^	0.795^**^	1		
ABC	−0.093	−0.020	−0.033	0.143	1	
AB	0.532^**^	0.570^**^	0.536^**^	0.573^**^	−0.065	1

**Notes.**

* Correlation is significant at *p* < 0.05.

** Correlation is significant at *p* < 0.01.

## Discussion

The proper functioning of the cranio-cervical neurovascular system depends on the odontoid process of C2, which enables safe movement of the head and neck. To diagnose and treat individuals with cranio-cervical spine problems, it is crucial to have an understanding of the morphometric values of the odontoid process. Previous studies have used morphometric analysis of the odontoid process to investigate screw insertion for odontoid process fractures (Korres et al., 2016; Naderi et al., 2006; Bakirci, Sendemir & Kafa, 2014; Kulkarni et al., 2013; Sharma et al., 2015). However, this paper proposes a novel approach using CBCT data applications to examine odontoid process morphometrics in the Arab population of the UAE, which to our knowledge is the first of its kind. In cases of basilar invagination and brain stem compression, an increase in the length of the dens may cause upward bulging into the foramen magnum and compress the brainstem, which underscores the importance of studying the morphometric values of the odontoid process. Odontoid process fractures are common and account for 10–15% of all cervical spine fractures. Patients who are younger than 8 years old or older than 70 years old have a higher likelihood of experiencing odontoid process fractures compared to other types of cervical fractures (Kyoshima et al., 2005; Devlin, 2020). Korres et al. (2008) categorized odontoid fractures into four types: type A, which involves the tip of the dens and has an incidence rate of 2.3%; type B, which occurs at the neck and has an incidence rate of 44%; type C, which involves the base of the dens and is the most common type of fracture with an incidence rate of 46.6% (including C1 and C2); and type D, which is a complex fracture and has an incidence rate of 7%. The majority of odontoid fractures can heal without surgery, but unstable fractures that may result in pseudarthrosis will require surgery (Korres et al., 1989). The optimal treatment outcome for a broken odontoid process in the cervical spine should involve pain relief, stability, and as much natural range of motion as possible. This can be achieved through osteosynthesis of the fractured odontoid process using one or two screws, depending on the size of the odontoid process. Fractures of types B and C1 can be stabilized using screws  (Korres et al., 2016). In order to achieve maximum stability, it is important to understand the anatomy and dorsal diversion of the odontoid process to ensure optimal screw placement (Tun et al., 2009). The number of screws required, as well as their length and diameter, depend on the size of the odontoid process. This study used precise and reliable measuring techniques, as demonstrated by high ICCs indicating measurement repeatability, to examine odontoid process morphology through CBCT-based evaluation techniques. There is ongoing debate regarding the practicality of using multiple screws for stabilization (Nucci et al., 1995; Yusof et al., 2007; McBride et al., 1995). It is generally agreed that a transverse diameter of at least 9.0 mm is needed to accommodate two screws with a diameter of 3.5 mm securely (Bakirci, Sendemir & Kafa, 2014). Reported odontoid process morphometry varies among different populations, and our data revealed that 97 (67.4%) participants did not have a METDs of >9 mm, indicating that only one screw would be needed to stabilize their fracture. Our findings suggest that a 4.5-mm Herbert screw can be inserted with ease, even in female patients, which is consistent with existing literature (Ece, Atalar & Gulekon, 2019). However, 48 individuals (31.83% of the sample) had space for two 3.5 mm or two 2.7 mm screws. While two 3.5 mm screws were suitable for 95% of Caucasian odontoid processes (Nucci et al., 1995), this was only the case for 33% of Malaysian odontoid processes (Nucci et al., 1995), 35% of Brazilian odontoid processes (Daher et al., 2011), 89.1% of the Greek population (Korres et al., 2016), and 70% of the Turkish population (Ece, Atalar & Gulekon, 2019). In this study, we found that the length of the odontoid process (AB) ranged from 31 to 46 mm in males and 31 to 40 mm in females. To ensure maximum stability, the length of the screw used must be 1–3 mm shorter than the actual length of the odontoid process. The screw thread must also match the inner diameter of the odontoid medulla, which has not been previously reported in the literature (Korres et al., 1989). Our study found that the inner diameter of the odontoid is 6.18 ± 0.78 mm for males and 5.87 ± 0.73 mm for females, and the screw thickness should be near this value. The appropriate screw diameter must be determined using axial CT for each case, and the length of the screw must be precisely measured to ensure proper fixation and compression. The length of the odontoid process measured in our study was comparable to that of a previous study by Puchwein et al. for both genders. The goal of the anterior odontoid screw method is to drag the broken odontoid fragment against the body of the axis for proper instrumentation. Fracture healing is significantly improved by applying pressure to the cracked edges and stabilizing the fracture. Therefore, it is crucial to assess screw length prior to surgery. Short screws are inadequate as they cannot cross the fracture line, penetrate the tip of the odontoid, or draw the broken odontoid piece (Puchwein et al., 2013). Conversely, lengthy screws may penetrate the odontoid tip, causing harm to the vertebral artery and neural tissue (ElSaghir & Böhm, 2000). The current study’s selected linear measurements show a strong positive correlation, indicating that they are the most effective parameters for detecting odontoid process morphometry. Using CBCT-reconstructed images, the diameter and length of the odontoid process and an estimate of bone quality and size can be calculated to ensure the safety and efficacy of one-to-two screw fixation. Imaging with CBCT has developed as a cost-effective technique for assessing the odontoid process. In comparison to MDCT, CBCT imaging gives high-quality, detailed pictures for a lower cost. CBCT imaging provides the added benefit of less radiation exposure, making it a safer alternative for patients. Considering its precision, speed, and affordability, CBCT imaging has the potential to replace more costly MDCT for odontoid process assessments, making it a helpful tool for healthcare providers seeking to maximize patient treatment while minimizing expenses (DeNunzio et al., 2022). The ABC angle is a novel measurement that represents the angle formed between the line connecting the apex of the odontoid process to the anterior edge of the axis and the tangent to the plateau below the axis. This measurement has not been previously reported in the scientific literature. Our study using CBCT showed that the ABC angle was 63.28 ± 3.94° for males and 62.48 ± 3.46° for females. Surgeons should take into consideration the ABC angle during surgery. If the odontoid has a larger angle, it may be necessary to rim the superior anterior part of the third cervical vertebra to accommodate the screwdriver. However, if the odontoid has a lower angle, the screw can be placed without rimming. It is important for surgeons to be aware of factors that may limit the application of this technique, such as concomitant thoracic kyphosis, short neck, barrel chest deformity, and fracture configurations that require a flexed position to obtain and maintain reduction. These factors can prevent hyperextension and impede the ideal screw trajectory during positioning (Reindl, Sen & Aebi, 2003).

The Arab population’s ancestry is diversified, with origins in both Asia and Africa. This ethnic variance may affect the accuracy of measures obtained in studies with Arab participants and might be regarded a possible limitation of this study. In order to conduct a more precise analysis, future research will need a larger sample size and the separation of Arabs according to their ancestry.

## Conclusion

About sixty percent of the sample had METDs of less than nine millimeters, indicating that a single 4.5-mm Herbert screw may be recommended for repairing fractured odontoid processes in the Arab population.

Individual CBCT readings facilitate preoperative operations including the determination of the inner odontoid diameter and the screw angle. It is necessary to measure the screw length utilizing 3-D CT or CBCT to have a better knowledge and visual depiction of the screwing phase throughout the process. This information makes patient positioning on the surgical table easier and more precise.

##  Supplemental Information

10.7717/peerj.15411/supp-1 Supplemental Information 1 Anonymized raw data

## Additional Information and Declarations

### Data Availability

The following information was supplied regarding data availability: The raw data is available in the Supplementary File.
